# Descriptive epidemiology of cholera outbreak in Nigeria, January–November, 2018: implications for the global roadmap strategy

**DOI:** 10.1186/s12889-019-7559-6

**Published:** 2019-09-13

**Authors:** Kelly Osezele Elimian, Anwar Musah, Somto Mezue, Oyeronke Oyebanji, Sebastian Yennan, Arisekola Jinadu, Nanpring Williams, Adesola Ogunleye, Ibrahima Soce Fall, Michel Yao, Womi-Eteng Eteng, Patrick Abok, Michael Popoola, Martin Chukwuji, Linda Haj Omar, Eme Ekeng, Thieno Balde, Ibrahim Mamadu, Ayodele Adeyemo, Geoffrey Namara, Ifeanyi Okudo, Wondimagegnehu Alemu, Clement Peter, Chikwe Ihekweazu

**Affiliations:** 1 Nigeria Centre for Disease Control, Abuja, Nigeria; 2 University of Benin, Benin City, Edo State Nigeria; 3 University College London, London, UK; 4 World Health Organization/ Regional Office for Africa, Brazzaville, Democratic Republic of Congo; 5 World Health Organization/ Nigeria, Abuja, Nigeria; 6 eHealth Africa, Abuja, Nigeria

**Keywords:** Cholera, Outbreak, Attack rate, Case fatality rate, Global roadmap, Nigeria, Multi-sectoral

## Abstract

**Background:**

The cholera outbreak in 2018 in Nigeria reaffirms its public health threat to the country. Evidence on the current epidemiology of cholera required for the design and implementation of appropriate interventions towards attaining the global roadmap strategic goals for cholera elimination however seems lacking. Thus, this study aimed at addressing this gap by describing the epidemiology of the 2018 cholera outbreak in Nigeria.

**Methods:**

This was a retrospective analysis of surveillance data collected between January 1st and November 19th, 2018. A cholera case was defined as an individual aged 2 years or older presenting with acute watery diarrhoea and severe dehydration or dying from acute watery diarrhoea. Descriptive analyses were performed and presented with respect to person, time and place using appropriate statistics.

**Results:**

There were 43,996 cholera cases and 836 cholera deaths across 20 states in Nigeria during the outbreak period, with an attack rate (AR) of 127.43/100,000 population and a case fatality rate (CFR) of 1.90%. Individuals aged 15 years or older (47.76%) were the most affected age group, but the proportion of affected males and females was about the same (49.00 and 51.00% respectively). The outbreak was characterised by four distinct epidemic waves, with higher number of deaths recorded in the third and fourth waves. States from the north-west and north-east regions of the country recorded the highest ARs while those from the north-central recorded the highest CFRs.

**Conclusion:**

The severity and wide-geographical distribution of cholera cases and deaths during the 2018 outbreak are indicative of an elevated burden, which was more notable in the northern region of the country. Overall, the findings reaffirm the strategic role of a multi-sectoral approach in the design and implementation of public health interventions aimed at preventing and controlling cholera in Nigeria.

**Electronic supplementary material:**

The online version of this article (10.1186/s12889-019-7559-6) contains supplementary material, which is available to authorized users.

## Background

Cholera is an acute watery diarrhoeal disease caused by the ingestion of food or water contaminated with the toxigenic strains of *Vibrio cholerae* serogroups O1 or O139 [1]. Cholera is often characterised by watery diarrhoea, with or without vomiting, and severe dehydration, resulting in death if left untreated [2]. The Case Fatality Rate (CFR) from untreated cholera can be as high as 30–50%, but prompt administration of rehydration therapy can reduce it to as low as 1% [2]. The global estimates for cholera cases and deaths are about 2.9 million and 95,000 per year, respectively [3], disproportionately affecting sub-Saharan African countries especially since onset of the seventh pandemic in 1961 [1]. For instance, 17 African countries reported over 150,000 cholera cases from all the outbreaks in 2017.

Historically, Nigeria has experienced several cholera outbreaks characterised by high CFRs, notable ones being the epidemic of 1991 which resulted in 59,478 cases and 7654 deaths, and the CFR of 12.9% reported for that outbreak remains the highest for the country to date. Furthermore, another major cholera outbreak occurred in Kano state in March, 1999, with cases spreading to Adamawa and Edo states by May of that year; and the outbreak resulted in 26,358 cases and 2085 deaths. From January to December 2010, Nigeria reported 41,787 cases and 1716 deaths (CFR 4.1%) across 18 states [4]. The last major cholera outbreak prior to 2018 was in 2014, during which the number of cases recorded cases surpassed over half of the number of cases recorded between 2012 and 2013 as well as between 2015 and 2017. In line with global evidence, however, it is likely that cholera burden in Nigeria is underestimated due to factors ranging from differences in case definitions and completeness to social, political, and economic disincentives for reporting cholera [5]. Nonetheless, in response to the increasing global cholera burden, the Global Task Force on Cholera Control (GTFCC), in 2017, launched the Global Roadmap Strategies which seek to reduce cholera-related deaths by 90% as well as eliminate cholera infections in at least 20 out of the 47 endemic countries by 2030 [6]. Nigeria has taken fundamental steps toward attaining these goals by deploying Oral Cholera Vaccines (OCVs) in cholera hotspots. Since the first deployment in September 2017 to date, million doses of OCVs have been deployed, albeit in a reactive context, across several hotspot areas, predominantly in the northern states (e.g. Borno, Bauchi, Yobe and Adamawa states) of Nigeria. Also in line with the GTFCC recommendations, Nigeria is finalising its National Strategic Plan of Action on Cholera Control. Despite the aforementioned efforts toward cholera prevention and control, the cholera outbreak of 2018 however reaffirms the serious public health threat of cholera and, importantly, the need for the country to adopt holistic countermeasures.

In brief, a surge in reported diarrhoea cases among adults in Kano and Kaduna states towards the end of 2017 raised a suspicion of cholera, prompting an epidemiological investigation by a rapid response team. The investigation involved using rapid diagnostic tests and microbiological investigations of stool samples or rectal swabs for diagnosis as per the Nigeria Centre for Disease Control (NCDC) guidelines [7]. Upon confirmation of *V. cholerae* in line with the NCDC guidelines, cholera outbreak was declared on January 1, 2018. Health facilities at various levels of care (primary, secondary, tertiary, and specialised units such as Cholera Treatment Centres (CTCs)) in affected Local Government Areas (LGAs) relied on rapid diagnosis for subsequent testing of suspected cholera cases, but sending stool samples to the NCDC reference laboratory in Abuja for confirmatory test. In line with the Integrated Disease Surveillance Response (IDSR) system, all the reported cholera cases were collated and submitted by each reporting health facility to the Disease Surveillance and Notification Officer (DSNO) with the aid of predefined line-lists; the data were transmitted by the DSNO on a weekly basis to the State Epidemiologist for aggregation and further transmission to the Surveillance and Epidemiology Department (SED) at NCDC in Abuja, where basic statistical analyses and disseminations of findings to reporting states are undertaken. The National Cholera Emergency Operations Centre was de-escalated to a Technical Working Group on November 20, 2018, but with continued monitoring and support to affected states. Indeed, the 2018 cholera outbreak represents an opportunity to re-assess how well and how far Nigeria is progressing towards attaining global roadmap strategic goals, and to provide current epidemiology of cholera in the country, with a view to providing the evidential-basis for a holistic public health planning and effective interventions. For example, identifying the most affected age group and areas, especially in the context of emerging cholera hotspots in the country, will be useful for an efficient allocation of limited resources towards cholera prevention and control. To this end, this study describes the epidemiology of the 2018 cholera outbreak in Nigeria in terms of time, place and person.

## Methods

### Study approach, period, and setting

This was a retrospective analysis of secondary surveillance data spanning between January 1st and November 19th, 2018. The 20 states affected by the outbreak and their corresponding geopolitical zones were: Anambra and Ebonyi (south-east); Adamawa, Borno, Bauchi, Gombe and Yobe (north-east); Abuja, Kogi, Kwara, Nasarawa, Niger and Plateau (north-central); and Jigawa, Kaduna, Kano, Katsina, Kebbi, Sokoto and Zamfara (north-west).

### Data source and management

**Fig. 1:**
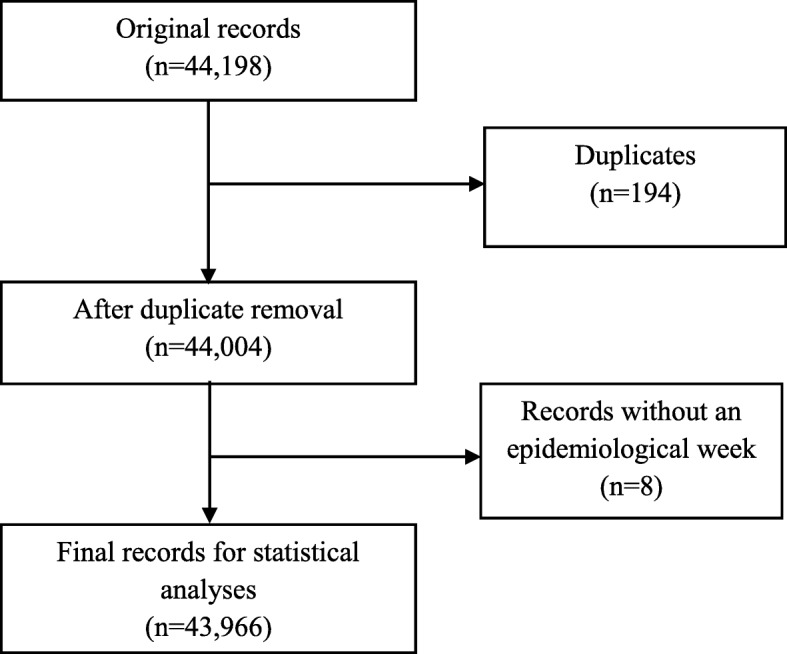
The Selection Process of Study Records, January 1st-November 19th, 2018

Secondary surveillance data in MS Excel format from the NCDC SED (primarily mandated for the coordination of cholera outbreak surveillance and response activities in Nigeria) was exported into Stata version 15 (StataCorp LP, College Station, TX, USA) for management and organisation. In line with NCDC ethical guidelines, specifically with respect to confidentiality and anonymity, all forms of identifiers (e.g. names, addresses, and telephone numbers) were deleted prior to data management. The processes for selecting the final dataset is shown in Fig. 1.

### Study population, and definition of key variables

The study population comprised individuals classified as having suspected cholera (herein: cholera cases) during the outbreak period. In accordance with the NCDC guidelines for preparedness and response to acute watery diarrhoea outbreak [7], a cholera case was defined as the detection of a cluster of persons aged 2 years or older with acute watery diarrhoea and severe dehydration or dying from acute watery diarrhoea from the same area within 1 week. In line with best practice in the context of a cholera outbreak [4], however, children under the age of 2 years who met the case definition were included in the current study as cholera cases. A confirmed cholera was defined as a cholera case in which *Vibrio cholerae* O1 or O139 was isolated in the stool by microbiological investigation [7]. The definition of other key study variables is shown in Table 1.

**Table 1:** Definition of key study variables

Variable	Definition
Epidemiological week	The first epidemiological week [herein: week] was defined as the week ending on the first Saturday of January 2018; subsequent weeks however began on Sunday and ended on Saturday. The current study covered week 1 to 47 out of the 52 weeks.
Epidemiological wave	In line with 2018 epidemiological weeks and a previous study [4], an epidemiological wave (herein: wave) was defined as the time between the commencement of a peak (first week with marked increase in the numbers of reported cases) and the end of a peak (epidemiological week marked decrease in the number of reported cases before the next rise in reported cases). The variable was classified as a categorical: first wave (between week 1 and week 9), second wave (between week 10 and week 28), third wave (between week 29 and week 34), and fourth wave (between week 35 and week 47).
Age	Age was defined in years and presented as a categorical variable.
Season	Dry season was defined as the period between week 1 and 12 and week 45 to 47, while rainy season was defined as the period between week 13 and 44.
Time to health seeking	This was defined as the difference between the reported date of health seeking and reported date of illness onset. It was classified as a categorical variable: same day, 1–2 days, and more than 2 days.
Location health care was sought	This was defined as primary healthcare centre, secondary hospital, tertiary hospital, cholera treatment centre (in any of the aforementioned health facilities), private clinic, and home. Information for its classification was validated by the DSNOs or State Epidemiologists of each affected state.
Outbreak setting	The affected LGAs were classified as rural, peri-urban or urban, using criteria of the population division of the United Nations which classifies an urban area as a settlement with 20,000 or more inhabitants, of which 75% or more are engaged in work other than agriculture, and a rural area as a settlement with fewer than 20,000 inhabitants whose primary occupation is agriculture. However, in the absence of a standard classification scheme, we classified a peri-urban area as a transition zone that is neither urban nor rural in the traditional sense. The provisional classifications were then validated using the respective state DSNOs’ or Epidemiologists’ final classifications.
Hospitalization	Hospitalization was defined as the admission of a cholera case to a formal health facility for at least one night.
Cholera death	Cholera death was defined as death of an individual classified as having cholera case in line with the case definition in the NCDC guidelines.
Attack Rate (AR)	AR was defined as the ratio of cholera cases in a defined area (e.g. state) to the estimated population of that area. AR for each reporting state was calculated using the estimated population of 2018, which was based on a 3.3% projected growth rate from the 2006 national census results; the values were multiplied by 100,000 for easier interpretation of small values.
Case Fatality Rate (CFR)	CFR was defined as the ratio of individuals classified as cholera cases who die to all those classified as cholera cases (alive and dead). CFR was expressed in percentage (%).

### Statistical analyses

Exploratory analysis of baseline characteristics of the study population was conducted using appropriate statistical summaries including frequency and percentage for binary/categorical variables, and mean and standard deviation for normally distributed continuous variables. Primary outcomes included attack rate (AR) and case fatality rate (CFR), and were presented with respect to other key study variables (Table 1). The standard approach to the description of a disease outbreak in terms of person, place and time was then used for further analyses. Similar to data management, all statistical analyses were performed in Stata version 15, and a *p*-value of less than 0.05 was considered statistically significant.

## Results

### Description of the study population

**Fig. 2:**
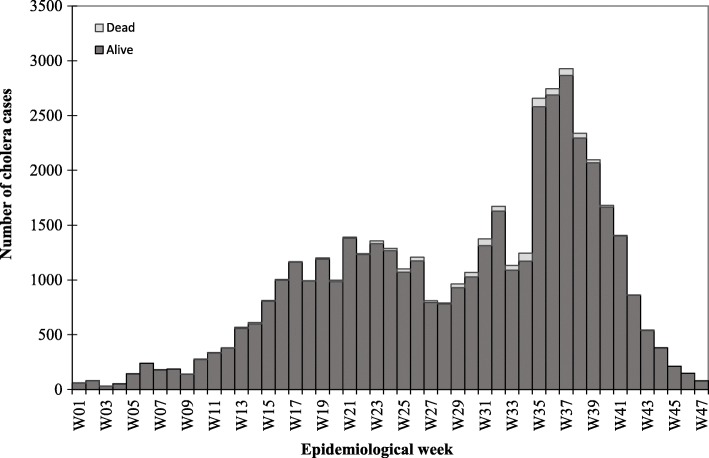
Reported cholera cases and deaths by epidemiological week, wk01–47, 2018

**Fig. 3:**
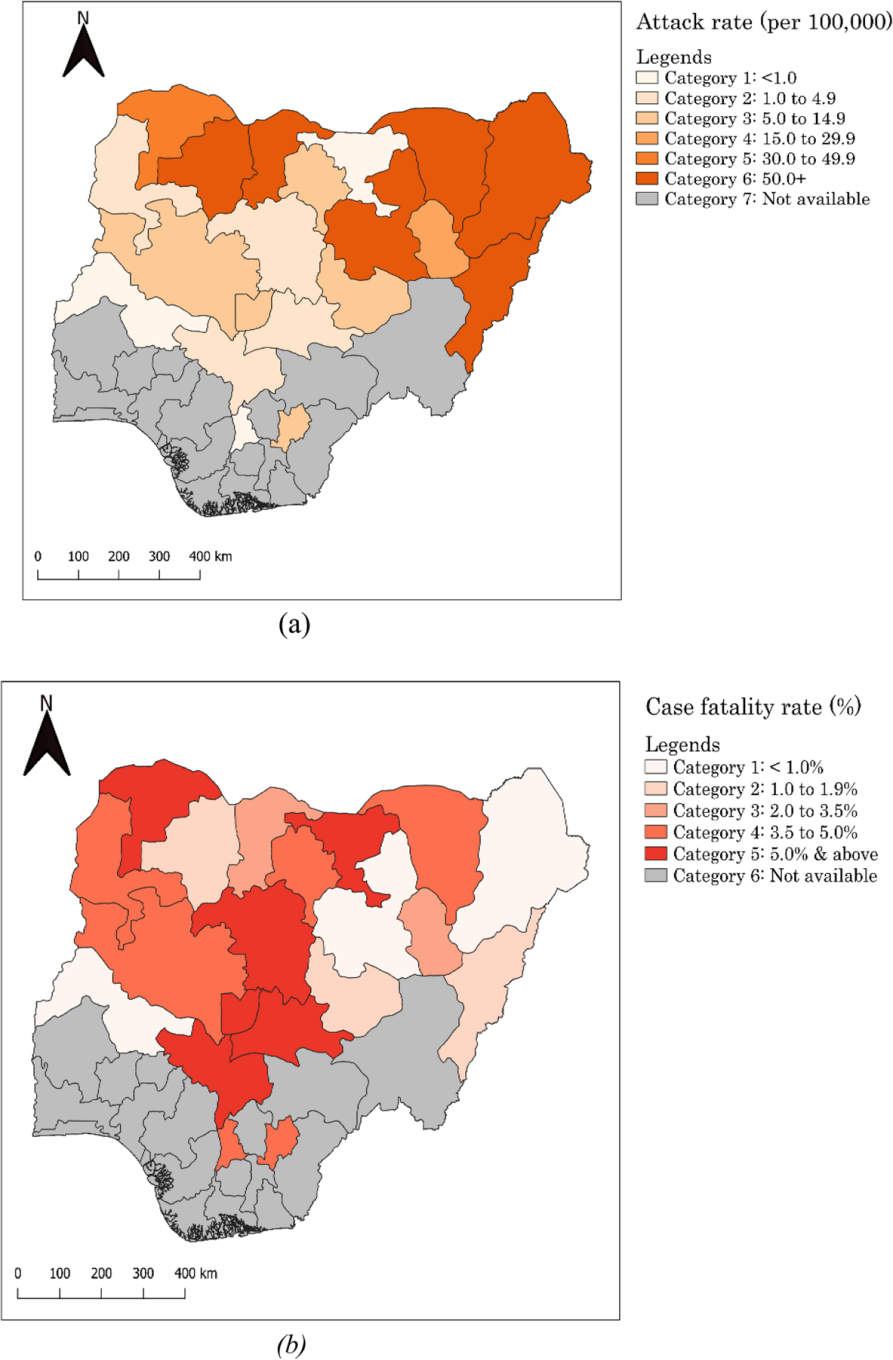
Spatial distribution of cholera in terms of attack rates (a) and case fatality rates (b). Map generated using QGIS version 3.2.3 software

Twenty out of 36 states (plus the federal capital city of Nigeria, Abuja) were affected by the cholera outbreak, resulting in a total of 43,996 cases and 836 deaths during the outbreak period. Figure 2 shows the epidemiological curve for cholera cases and deaths by epidemic week. The outbreak was characterised by four distinct epidemic waves and mirrored a propagated epidemic pattern, suggesting a person-to-person transmission. Notably, the majority of cholera cases occurred in the second and fourth waves, with a peak at week 37; there was however a preponderance of cholera deaths towards the end of the third wave and beginning of the fourth wave, with sporadic cases of death in between the two waves. As expected, the majority (94.48%) of cholera cases were reported during the rainy season. It was interesting to note that health care was sought within a day of illness onset by the majority of cases (62.44%), with a particular preference for primary healthcare facilities (19.24%) and secondary hospitals (14.31%). The number of samples tested by culture was extremely low as only 137 (12.03%) out of 1139 samples collected during the outbreak period were examined, yielding 92 confirmed cholera cases of serogroup O1, biotype El Tor and serotype Inaba (results not shown). The spatial distribution of cholera cases across the 20 affected states is shown in Fig. 3. The remaining sections of the paper are presented in terms of cholera distribution by person and place, and time.

### Distribution of cholera cases and deaths by person and place

Although the median age for the entire study population was 14 years (IQR: 5–30), individuals aged 15 years or older (47.76%) were the most affected age group during the outbreak (Table 2). With respect to gender, there was a slight dominance of females (50.74%) over males (49.26%). States from the north-east region of Nigeria accounted for over half (51.38%) of cholera cases, while those from the south-east region (0.47%) accounted for the least proportion of cases.

**Table 2:** Baseline characteristics of cases during the 2018 cholera outbreak in Nigeria (*N* = 43,996)

Baseline characteristic	Frequency (%)
Sociodemographic characteristic	
Sex	
Female	22,322 (50.74)
Male	21,674 (49.26)
Median (IQR) age, years^a^	14 (5–30)
Age, years	
< 2	2602 (5.91)
2–4	7651 (17.39)
5–10	8068 (18.34)
10–14	3300 (7.50)
> 15	21,014 (47.76)
Missing	1361 (3.09)
Geopolitical zone	
South-east	205 (0.47)
North-central	1335 (3.03)
North-west	19,850 (45.12)
North-east	22,606 (51.38)
Epidemiological wave	
First wave (week 1–9 of 2018)	1119 (2.54)
Second wave (week 10–28 of 2018)	17,528 (39.84)
Third wave (week 29–34 of 2018)	7454 (16.94)
Fourth wave (week 35–47 of 2018)	17,895 (40.67)
Season	
Dry	2427 (5.52)
Rainy	41,569 (94.48)
Outbreak setting	
Rural	15,501 (35.23)
Peri-urban	5941 (13.50)
Urban	22,077 (50.18)
Missing	477 (1.08)
Clinical characteristic	
Time to healthcare seeking	
Same day	27,470 (62.44)
1–2 days	10,159 (23.09)
> 2 days	1695 (3.85)
Missing	4672 (10.62)
Location healthcare was sought	
Primary healthcare centre	8464 (19.24)
Secondary hospital	6294 (14.31)
Tertiary hospital	58 (0.13)
Private clinic	143 (0.33)
Cholera treatment centre (including IDP camps)	730 (1.66)
Home	9 (0.02)
Missing	28,298 (64.32)
Hospitalisation	
No	6096 (13.86)
Yes	20,224 (45.97)
Missing	17,676 (40.18)
Stool sample collected for microbial test	
No	21,597 (49.09)
Yes	1139 (2.59)
Missing	21,260 (48.32)
Stool sample tested for *V. cholerae*	
No	42,928 (97.57)
Yes	1068 (2.43)
Rapid diagnostic test outcome	
Negative	205 (0.47)
Positive	888 (2.02)
Pending	3 (0.01)
Unknown	42,900 (97.51)
Culture outcome	
Negative	41 (0.09)
Positive	92 (0.21)
Pending	4 (0.01)
Unknown	43,859 (99.69)
Clinical outcome	
Alive	43,160 (98.10)
Died	836 (1.90)

^a^*IQR* Interquartile range

The overall attack rate during the outbreak period was 127.43/100,000 (Table 3); specifically, Zamfara (175.08/100,000 population) and Bauchi (134.65/100,000) states recorded higher ARs compared to other states such as Jigawa with an AR of 0.24/100,000 population. CFRs were generally high across all affected states, with about 70% of these states recording higher CFRs above the national figure of 1.90%. Notably, states from north-central recorded the highest CFRs [7.84% in Kogi and 7.04% in Nasarawa]. A sub-analysis of the data indicates that patients’ age and sex were significantly associated with CFR (Additional file 1), such that individuals aged 5 years or older recorded a higher CFR (2.11%) as compared with those under the age of 5 years (1.43%). In addition, males were found to record a higher CFR (2.12%) than females (1.69%).

**Table 3:** Distribution of cholera attack rates and case fatality rates by state, Nigeria, 2018

State	Projected 2018 population	Cases	Deaths	Attack rate/100,000 population	CFR (%)
Adamawa	4,464,609.877	2748	41	61.55	1.50
Anambra	5,825,118.003	23	1	0.40	4.35
Bauchi	6,984,963.699	9405	35	134.65	0.37
Borno	6,200,395.472	7626	74	123.00	0.97
Ebonyi	3,027,451.68	182	7	6.01	3.85
FCT^a^	4,084,890.258	221	14	5.41	6.33
Gombe	3,435,108.839	552	18	16.07	3.26
Jigawa	6,128,283.561	15	1	0.24	6.67
Kaduna	8,649,466.817	401	25	4.64	6.23
Kano	13,854,062.42	1905	73	13.75	3.83
Katsina	8,258,831.146	7400	190	89.60	2.57
Kebbi	4,671,593.545	198	7	4.24	3.54
Kogi	4,674,338.533	102	8	2.18	7.84
Kwara	3,380,605.955	10	0	0.30	0.00
Nasarawa	2,656,584.616	71	5	2.67	7.04
Niger	5,900,257.11	584	29	9.90	4.97
Plateau	4,376,193.378	347	6	7.93	1.73
Sokoto	5,271,036.573	1602	84	30.40	5.24
Yobe	3,508,083.395	2275	83	64.85	3.65
Zamfara	4,757,222.358	8329	135	175.08	1.62
Total	34,524,321.92	43,996	836	127.43	1.90

^a^ Federal Capital Territory of Nigeria (Abuja)

#### Time (epidemiological wave)

Higher number of cholera cases was recorded consistently in individuals aged 5 years or older throughout the outbreak period, particularly in the third wave during which they accounted for 77.06% of recorded cases (Table 4). However, the distribution of cholera cases was about even between males and females across all waves. With respect to geographical distribution, states from the north-east accounted for a higher number of cases in the first (78.11%) and second (70.58%) waves, whereas those from the north-west accounted for a higher number of cases in the third (86.87%) and fourth (51.70%) waves. In addition, the majority of cases in the first, third and fourth waves were recorded in rural areas. Time to health seeking was generally impressive across the four waves given that most individuals sought health care within a day of illness onset, particularly in primary health and secondary facilities; however, nine patients reported practising home-based management of illness in the second and fourth waves. Also notable was the very few records of deaths recorded in the first wave, following which the remaining decedents spread almost evenly across the second (*n* = 233), third (*n* = 296), and fourth (*n* = 295) waves.

**Table 4:** Distribution of cholera cases by epidemiological wave, Nigeria, 2018

Characteristic	Epidemiological wave				
	First wave Cases (%)	Second wave Cases (%)	Third wave Cases (%)	Fourth wave Cases (%)	Total Cases (%)
Age (years)					
< 5	386 (34.50)	4551 (25.96)	1422 (19.08)	3899 (21.79)	10,258 (23.32)
≥ 5	731 (65.33)	12,955 (73.91)	5744 (77.06)	12,947 (72.35)	32,377 (73.59)
Missing	2 (0.18)	22 (0.13)	288 (3.86)	1049 (5.86)†	1361 (3.09)
Sex					
Female	567 (50.67)	8782 (50.10)	3773 (50.62)	9200 (51.41)	22,322 (50.74)
Male	552 (49.33)	8746 (49.90)	3681 (49.38)	8695 (48.59)NS	21,674 (49.26)
Geo-political zone					
South-east	3 (0.27)	188 (1.07)	14 (0.19)	0 (0.00)	205 (0.47)
North-central	3 (0.27)	1083 (6.18)	158 (2.12)	91 (0.51)	1335 (3.03)
North-west	239 (21.36)	3885 (22.16)	6475 (86.87)	9251 (51.70)	19,850 (45.12)
North-east	874 (78.11)	12,372 (70.58)	807 (10.83)	8553 (47.80)†	22,606 (51.38)
Season					
Rainy	0 (0.00)	997 (5.69)	7454 (100.00)	311 (1.74)	2427 (5.52)
Dry	1119 (100.00)	16,531 (94.31)	0 (0.00)	17,584 (98.26)†	41,569 (94.48)
Outbreak setting					
Rural	887 (79.27)	3073 (17.53)	4093 (54.91)	7448 (41.62)	15,501 (35.23)
Peri-urban	53 (4.74)	918 (5.24)	1333 (17.88)	3637 (20.32)	5941 (13.50)
Urban	146 (13.05)	13,464 (76.81)	1985 (26.63)	6482 (36.22)	22,077 (50.18)
Missing	33 (2.95)	73 (0.42)	43 (0.58)	328 (1.83)†	477 (1.08)
Location care was sought					
Primary healthcare centre	52 (4.65)	656 (3.74)	2118 (28.41)	5638 (31.51)	8464 (19.24)
Secondary hospital	7 (0.63)	5219 (29.78)	390 (5.23)	678 (3.79)	6294 (14.31)
Tertiary hospital	1 (0.09)	8 (0.05)	14 (0.19)	35 (0.20)	58 (0.13)
Private clinic	0 (0.00)	107 (0.61)	29 (0.39)	7 (0.04)	143 (0.33)
Cholera treatment centre*	0 (0.00)	19 (0.11)	28 (0.38)	683 (3.82)	730 (1.66)
Home	0 (0.00)	5 (0.03)	3 (0.04)	1 (0.01)	9 (0.02)
Missing	1059 (94.64)	11,514 (65.69)	4872 (65.36)	10,853 (60.65)†	28,298 (64.32)
Time to health seeking					
Same day	862 (77.03)	8118 (46.31)	4691 (62.93)	13,799 (77.11)	27,470 (62.44)
1–2 days	50 (4.47)	6597 (37.64)	1622 (21.76)	1890 (10.56)	10,159 (23.09)
> 2 days	62 (5.54)	1252 (7.14)	216 (2.90)	165 (0.92)	1695 (3.85)
Missing	145 (12.96)	1561 (8.91)	925 (12.41)	2041 (11.41)†	4672 (10.62)
Hospitalised					
No	53 (4.74)	4418 (25.21)	334 (4.48)	1291 (7.21)	6096 (13.86)
Yes	81 (7.24)	8884 (50.68)	2073 (27.81)	9186 (51.33)	20,224 (45.97)
Missing	985 (88.03)	4226 (24.11)	5047 (67.71)	7418 (41.45)†	17,676 (40.18)
Clinical outcome					
Alive	1107 (98.93)	17,295 (98.67)	7158 (96.03)	17,600 (98.35)	43,160 (98.10)
Dead	12 (1.07)	233 (1.33)	296 (3.97)	295 (1.65)†	836 (1.90)

* Including Internally Displaced Persons’ camps

†*p*-value < 0.001; NS (Not Significant) =0.106; *p*-values cover the four epidemic waves

## Discussion

### Summary and interpretations of key findings

This study described the epidemiology of the 2018 cholera outbreak in Nigeria in terms of case and death distribution by person, place and time. Overall, there were 43,996 cholera cases and 836 deaths across 20 states. The AR and CFR during the outbreak were 127.43/100,000 population and 1.90%, respectively. The CFR of 1.9% in the current study is comparable with that for Africa at approximately 2% [5] but almost twice as low as the value recorded by Dalhat and colleagues during the 2010 cholera outbreak in Nigeria [4]. A CFR higher than the WHO recommended benchmark of < 1% [8, 9] is generally considered high and indicative of inadequate clinical case management or quality of care [1]. However, we think that the heterogeneity of cholera case definition in Nigeria could affect the precision of the estimated CFR in the current study [10], hence the need for caution in interpreting this finding. With respect to demographic characteristics of the study population, older age group was associated with increased cholera transmission during the outbreak, but gender bias with respect to cholera case distribution was not obvious. In general, the distribution of cholera cases by age and sex in Nigeria is dynamic given the mixed available evidence. For instance, some studies have reported higher number of cholera cases in adults than in children [11–16] and vice-versa [17, 18], and some studies have similarly reported higher number of cholera cases in females than in males [4, 12, 19] and vice-versa [20, 21].

Epidemiological waves during the cholera outbreak appeared to had been significantly influenced by seasonality. The marked increase in cholera cases and deaths between week 35 and week 37 (peak of the fourth wave) coincided with when the intensity of rainy season had begun to dwindle across the country, which could be explained by one or a number of factors: (1) many persons tend to rely more on unsafe water sources when water levels are decreasing towards the end of rainy season [22]; (2) there is an increased likelihood for water sources to be contaminated by floods around this period [22, 23]; and (3) *V. cholerae* survival tends to be enhanced by the synergistic effects of zooplankton on iron level concentrations in waterways around this period [24]. Furthermore, there is evidence to suggest that Islamic festivals (*Ramadan* and *Id el Kabir*) in 2018 might have played a role in the observed trends, notably in the second and fourth waves. The peak of the second wave coincided with the week after *Ramadan* had commenced, and the trend was maintained up to 2 weeks following the end of the festival. Also, initial phase of the fourth wave coincided with *Id el Kabir* period in 2018. It is therefore possible that the change in social behaviour during these periods might have influenced the observed trends in that the traditional gathering of families and relatives in large groups for meals, as well as increased chances for people to patronise street food and water vendors could potentially aid the transmission of cholera [25]. Nevertheless, in line with one of the GTFCC’s strategic axes (i.e. targeted multi-sectoral approach to prevent cholera recurrence), the current finding could serve as evidential-basis for a synergistic collaboration between public health and religious stakeholders in the design and implementation of cholera-focused interventions.

The potential impact of armed conflict on increased cholera burden as evidenced by the high number of cases in states from the north-east region of Nigeria is worth discussing. When water sources and waste management system are disrupted, as they have been during Boko-Haram insurgency activities in this region, the transmission of cholera is likely. This hypothesis is supported by studies in Yemen [26] and Liberia [27]. In addition, the high number of cholera cases in Bauchi (21.38%) in comparison to states directly affected by Boko Haram insurgency (e.g. 17.33% in Borno) could be explained by a ‘spill-over’ effect of armed conflict. The rapid influx of internally displaced persons from states directly affected by conflict to neighbouring states such as Bauchi could also create an enabling environment (e.g. inadequate or contaminated water sources, poor sanitation facilities, overcrowding, and limited capacity for healthcare delivery by health workers) for cholera transmission. This hypothesis is in line with the findings by Siddique and colleagues wherein poor living conditions of Rwandan refugees in Goma, Zaire, significantly increased cholera transmission [28]. Again, these findings are suggestive of the need to extend cholera stakeholders beyond the traditional public health actors. This would involve actively engaging the security agency such as the Nigerian military in planning, training and implementing cholera-focused interventions, particularly given the fact that security personnel are usually among the first responders to such contexts. The feasibility of such collaboration is worth exploring in a follow-up study. Flooding in 2018 appeared to have aided cholera transmission and associated CFRs, particularly in Anambra, Kogi and Niger states. Flooding generally disrupts access to essential commodities including safe water sources and health care delivery, thereby exacerbating the occurrence of cholera and adverse clinical outcomes such as deaths [29]. The potential impact of flooding on cholera deaths in Nigeria is not a new phenomenon as underlined in the 2010 outbreak [4].

The public health implications of Inaba serotype dominance in the 2018 outbreak is also worth mentioning given the dominance by Ogawa serotype for long period in Nigeria. For instance, the Ogawa serotype of *V. cholerae* was in circulation during the 2010 cholera in Nigeria as evidenced by the molecular characterisation of samples from Borno, Bauchi and Gombe states [30]. Unlike the Ogawa serotype which appears to have established its niche in diverse contexts across Nigeria, the Inaba serotype appears to be localised in northern Nigeria such as Kano, Bauchi and Plateau states [31–33]. This could potentially explain why the 2018 cholera appeared to have originated from communities in Kano state; and why there were more cholera cases in adults than in children under the age of 5 years in the current study as evidenced by a study conducted in Bangladesh [34], albeit further evidence is required for a thorough explanation. Nevertheless, in the absence of a concrete evidence as to how and why *V. cholerae* serotypes change, the dominance of Inaba serotype in the 2018 outbreak could be attributable to ‘natural conversion as part of survival mechanism [34, 35]. Also, we do not think the severity of the 2018 cholera outbreak compared with previous years is related to the dominance of Inaba serotype as the two serotypes are similar pathogenicity [36, 37].

### Study strengths and limitations

The current study has the advantage of using a more representative data than similar studies in Nigeria, as all the 20 affected states were captured in the analyses. In describing the epidemiology of the 2010 cholera outbreak in Nigeria, for example, Dalhat and colleagues utilised data from only 10 out of the 18 affected states that submitted surveillance data to the ministry of health [4]. Given the current nature and scope of our study, the findings will be useful in developing prevention and control measures towards attaining the global roadmap strategic goals. Scientifically, the findings will also be useful to public health researchers in assessing the impact of interventions such as OCV campaigns. Our study however has a number of potential limitations worth mentioning. The definition of a cholera case [7] is at variance with the WHO’s definition with respect to age – our definition uses 2 years as the benchmark while that of WHO uses 5 years, albeit children under five meeting the case definition for cholera in endemic areas are considered suspected cases in both definitions [38]. Pathogens other than *V. cholerae* could be responsible for cholera-like symptoms [39], which could potentially affect the precision of our findings as children under-5 years were included in the definition of suspected cholera cases in the current study. The lack of a homogenous definition of cholera especially in an endemic setting such as Nigeria made the direct comparison of our findings with similar studies challenging. For example, studies in Nigeria have used 5 years [12, 17, 40], 2 years [21, 41, 42], and no age restrictions [14, 19] for the definition of suspected cholera cases. Thus, this challenge should be prioritised by the GTFCC given the relevance to cholera case management and assessment of cholera burden, which would be strategic in the objective assessment of the global roadmap goals of cholera elimination by 2030.

## Conclusion

The severity and wide-geographical distribution of cholera cases and deaths during the 2018 outbreak are indicative of an elevated burden, which was more notable in the northern region of the country. Overall, the current distribution of cholera cases and cholera-related deaths by person, place and time reaffirms the strategic role of a multi-sectoral approach in the design and implementation of public health interventions aimed at preventing and controlling cholera in Nigeria.

## Additional file

Additional file 1: Table showing the age and sex distribution of case fatality rates among cholera cases, Nigeria, 2018. (DOCX 12 kb)

## Data Availability

The datasets used for this study are available from the corresponding author upon reasonable request.

## Abbreviations

AR: Stands for Attack Rate
CFR: Stands for Case Fatality Rate
DSNO: Stands for Disease Surveillance and Notification Officer
GTFCC: Stands for Global Task Force on Cholera Control
LGA: Stands for Local Government Area
NCDC: Stands for Nigeria Centre for Disease Control
SED: Stands for Surveillance and Epidemiology Department
