# Supplementary material for: Descriptive epidemiology of cholera outbreak in Nigeria, January–November, 2018: implications for the global roadmap strategy
Source: BMC Public Health. 2019 Sep 13;19:1264. doi: 10.1186/s12889-019-7559-6 (PMC6743111; doi:10.1186/s12889-019-7559-6)
Supplement: Supplementary file 1 — Table showing the age and sex distribution of case fatality rates among cholera cases, Nigeria, 2018. (DOCX 12 kb) [file 12889_2019_7559_MOESM1_ESM.docx]

| **S1 Table: Age and sex distribution of case fatality rates among cholera cases, Nigeria, 2018** | | |
| --- | --- | --- |
| **Characteristic** | **Cholera cases (n/%)** | **CFRs (%)** |
| **Age group (n=42,635)**  <5 years  ≥5 years | 10,258 (24.06)  32,377 (75.94) | 1.43  2.11 |
| **Sex (n=43,996)**  Female  Male | 22,322 (50.74)  21,674 (49.26) | 1.69  2.12 |
